# Medical and Philosophical Intelligence

**Published:** 1811-07

**Authors:** 


### MEDICAL AND PHILOSOPHICAL INTELLIGENCE.

*Procedings of the Royal Society of Sciences, at Harlem, for* 1810.—The Society held its 57th anniversary meeting, on the 19th of May. The Secretary reported the communications which the Society had received on the physical sciences since its last meeting May 20, 1809. From this report it appeared,

I. That the following papers were approved of for publication.

1. F. W. Freyer, additions to his memoirs upon the diseases of fruit-trees, which obtained the gold medal in 1808.

2. J. Buys, upon Echo, particularly that of Minderberg.

3. I. Logger, upon a remarkable ossification.

II. That a memoir in Dutch had been received too unsatisfactory to be crowned; on the following question: "Quels sont les changements que les grandes rivieres, pour autant qu'elles parcourent ce royaume, ont subi par elles-mêmes, et sans le secours de l'art, dans les deux ou trois derniers siècles, et que peut on deduire, soit pour corriger les defauts des rivieres, soit pour en eviter les accidens facheux?"

III. Upon the question, "Pourrait-on établir avec avantage, près de nos cotes maritimes pour rassembler du sel brut, des batimens qu'on nomme en Allemagne *gradeerhausen*, pour l'evaporation de l'eau de mer, et de quelle maniere pourrait on essayer dans ce cas une telle entreprize, selon les circonstances locales et particulières à ce pays?" Two memoirs were received with these mottos; No. 1, *Nut en Voordeel*, and No. 2, *Quidquid agis, prudenter agas et respice finem.* These answers, though possessed of merit, were judged unworthy of being crowned; and the question is again proposed, and is open for candidates till the 1st of January 1812, with the addition of 50 ducats to the usual prize.

IV. Upon the question, "Quels sont les insectes qui sont les plus nuisibles aux arbres fruitieres dans ce pays-ci? Que sait on de leur économie, de leur metamorphose, de leur generation et des circonstances qui favorisent leur multiplication ou qui s'opposent? Quels moyens peut on déduire de l'un et de l'autre comme les plus convenables pour les diminuer; et qu'els moyens connait-on, par des experiences, pour en garantir les arbres susdits?" a memorial in German, having for its motto, *In minimis ut fere nullis, quanta vis!* was deemed to contain a satisfactory answer, and to be worthy of the gold medal, with the additional prize of 30 ducats. On opening the billet, it appeared that the author was Friedrich Wilhelm Freyer, Advocate of the Court and of the regency at Saxen Hilburghausen.

V. Upon the question, "Jusqu'à quel point connait-on, après les derniers progrés que l'on a fait dans la physiologie des plantes, de quelle manière les differens engrais, pour differens terroirs, favorisent la vegetation des plantes, et quelles indications peut-on deduire des connaissances acquises sur ce sujet pour le choix des engrais et la fertilization des terroirs incultes et arides?" two memoirs in Dutch were received, No. 1 having for its motto, *Natuur door kunst en vlijt;* and No. 2, *Mest sterkt de plant.* These answers were deemed unsatisfactory from the want of theoretical theoretical principles, and the question stands over till the 1st of January, 1812, with the addition of 30 ducats to the usual prize.

The Society repeats the six following questions, to be answered before the 1st of January, 1812.

I. " Jusqu'à quel point l'étude des anciens auteurs latins et autres, l'examen des monumens antiques, et des observations faites dans les terrains, peuvent elles servir à déterminer avec certitude quelle a été ci-devant, et surtout sous la domination romaine, la face de ces pays, le cours des riviéres et l'étendue des lacs qui composent ce royaume, et quels changemens successifs ont-ils subis depuis ?"

The Society is desirous of seeing this subject examined again, and of its being precisely indicated what is known of it with certainty, and what ought to be considered as doubtful, in what celebrated authors have written upon it.

II. " Qu'est-ce que les relations historiques, dont l'authenticité est reconnue, nous apprennent des changemens qu'ont subi la côte maritime de la Hollande, les iles et les bras de mer qui les separent, et quelles instructions utiles peut-on tirer de ce qui en est connu ?"

III. " Les flux de nos côtes maritimes montent-ils actuellement à de plus grandes hauteurs que dans les siècles précédens, et les reflux descendent-ils proportionellement moins bas que ci-devant? S'il en est ainsi, jusqu'à quel point peut-on determiner la quantité de cette différence dans des siècles plus ou moins reculés, et quelles sont les causes de ces changemens ? Se brouvent-elles dans les alterations successives des embouchures, ou dependent-elles de causes exterieures et plus éloignées, et quelles sont ces causes ?"

The Society engages to add an extraordinary prize of 30 ducats to the customary medal, for the best answer, to each of the two first questions, and to add a prize of 50 ducats for the third.

IV. " Comme les experiences et les observations des physiciens du dernier temps ont fait voir, que la quantité d'air vital que les plantes exhalent, n'est nullement suffisante pour rétablir dans l'atmosphére tout l'air vital consommé par la respiration des animaux, par la combustion, par l'absorption, &c. ; on demande par quelles autre voies l'equilibre entre les parties constituantes de l'atmosphère est continuellement conservé ?"

V. " Jusqu'à quel point la chimie a-t-elle fait connaitre les principes ou parties constituantes, tant eloignées que prochaines des plantes, surtout de celles qui servent à la nourriture ? Et jusqu'à quel point peut-on deduire de ce qu'on en sait, ou en poura decouvrir par des experiences combinées avec la physiologie du corps humain, quelles plantes sont les plus convenables pour le corps humain dans l'état de santé et dans quelques maladies ?

The Society offers an extraordinary prize of 30 ducats, in addition to the ordinary one, for a satisfactory answer to this question.

VI. " Quelle est la cause de la phosphorescence de l'eau de mer dans les mers et les flux de mer qui se trouvent dans ce royaume et dans les mers affluentes ? Ce phénomène depend-il de la presence d'animalcules vivans ? Quels sont, dans ce cas, ces animalcules dans l'eau de mer, et peuvent ils communiquer à l'atmosphère des propriétés nuisibles à l'homme ?"

In

In considering this question, the candidate is requested to examine what relation the phosphorescence of the sea-water, which is very remarkable in some parts of these countries, bears to the prevailing diseases in the least healthy seasons. Those who intend to answer this question, are invited to consult the most recent and exact observations on the subject, and especially those of Viviani. 1805.

The Society proposes for this year the eight following questions, the prize-essays to be transmitted before the 1st of January, 1812.

I. Since it appears that the secretion of the milk of cows is increased when they are fed in stables on potatoes, carrots, or red-beet (betterave), it is asked,

1. " Qu'il soit démontré par des experiences et des observations, si le lait des vaches est réellement augmenté par les nourritures susdites, et dans quelles circonstances cette augmentation a lieu. 2. De quelle manière l'ont peut donner ces nourritures avec le plus de profit. 3. Si la qualité du lait est altérée par les nourritures, et en quoi consistent alors ces alterations en general, et particulièrement à l'égard de la qualité et de la quantité relative de crême et de beurre que le lait peut produire.

II. As the anti-septic quality of common salt does not appear to depend solely on the muriate of soda, but also on the muriate of magnesia which is attached to common salt, it is required to determine by experiments: 1. " Dans quelle proportion se trouve la qualité anti-septique des deux sels susdits. 2. Quelle est la proportion dans laquelle ces deux sels doivent être mêlés pour prevenir le plus long-temps la putréfaction, sans que le goût des substances que l'on veut conservir devienne moins agréable. 3. S'il y a des cas dans lequels il serait avantageux de se servir uniquement du muriate de magnesie, particulièrement dans les expeditions pour des contrées plus chaudes ?"

III. " Quelle est la cause chimique que la chaux de pierre fait sur le total une maçonnerie plus solide et plus durable que la chaux de coquilles, et quels sont les moyens de corriger à cet égard la chaux de coquilles ?

IV. " Pourrait on établir dans ce pays, avec profit, des salpétrières, surtout dans les lieux ou l'eau est impregnée de plusieurs substances produites par la putréfaction des corps animaux ? Et quelles régles aurait-on alors à observer à cet égard ?"

V. " Qu'y a-t-il connu par des observations incontestables, par rapport à la nature des météores lumineux ou qui ont l'apparence du feu à l'exception de la foudre, comme il en parait de temps en temps dans l'atmosphere ? Jusqu'à quel point peut-on les expliquer par des experiences connues ? Qu'est ce qu'il y a encore de gratuitement soutenu ou de douteux dans ce que les physiciens de nos jours en ont avancé ?"

VI. " Peut-on démontrer par des expériences incontestables, que les substances qui ont l'apparence des métaux et qui ont été produites par des sels alcalins, sont des vraies métaux ? Ou, y a-t il des raisons suffisantes pour soutenir que ce sont des hydrures, produites par la combinaison de l'hydrogéne avec les sels alcalins ? Quelle est la maniére la plus sûre et la plus convenable de produire ces substances des sels alcalins en une quantité assez considerable au moyen d'une haute temperature ?

VII. " Jusqu'à quel point peut-on soutenir encore la doctrine de Harvey, vey, que les animaux naissent en general par des œufs preexistans, et que les plantes ne viennent que par les graines? Et quelles sont, au contraire, les observations principales qui font voir qu'il y a des animaux et des plantes qui proviennent d'une maniere différente?"

VIII. "Quel jugement faut-il porter sur les explications chimiques qu'on a taché de donner des phénomènes électriques? Y en a-t-il qui sont fondés sur des experiences suffisantes, ou peut-on les prouver par des experiences nouvelles? Ou faut-il les regarder jusqu'ici comme des hypothéses nullement prouvées, ou posées sans des raisons valables?"

The Society adds to the ordinary medal of 30 ducats, a gratification of 30 ducats for a satisfactory reply to the questions No. II, III, V, VI, and VII.

The Society has proposed in the preceding years, the following questions to be answered before the 1st of January, 1812.

I. "Qu'est-ce que les dernières observations ont appris sur l'influence de l'oxigène de l'air atmosphèrique, soit combiné ou non avec l'action de la lumiére sur le changement des couleurs; et quels avantages peut-on en tirer?" The Society wishes that whatever has been proved by observation and experiment should be succinctly and precisely demonstrated, that the actual state of our knowledge on this subject may be more easily come at, and may be rendered more profitable, both in commerce and other branches of economy.

II. "Qu'y a-t-il de vrai de toutes ces indications concernant les saisons prochaines ou des changemens de temps, qu'on croit trouver dans les vol des oisseaux, dans le cri ou dans les sons qu'on entend à certains temps, soit des oisseaux ou des autres animaux, et en général dans ce qu'on observe de plusieurs genres d'animaux à cet égard? L'expérience a-t-elle fait voir dans ces pays-ci l'un ou l'autre assez souvent pour s'y fier? Qu'est-ce qui est au contraire encore douteux de ce qu'on prétend à cet égard, ou réfuté par l'expérience? Et jusqu'à quel point peut-on expliquer ce qu'on en a observé par ce qu'on connaît de la nature des animaux?" The society wishes to collect in one point of view, all that observation has shewn on this subject with respect to the particular animals of their own country, or such as are sometimes seen in France, that the explanation of the question may be advantageous to the inhabitants of both countries.

III. "Qu'est-ce que l'experience a suffisamment prouvé concernant la purification de l'eau corrompue et d'autres substances impures, au moyen du charbon de bois? Jusqu'à quel point peut-on expliquer, par des principes de chimie, la manière dont elle se fait? Et quels avantages superieurs peut-on en tirer?"

IV. "Quelle est la différence réelle des propriétés et des principes ou parties constituantes du sucre, tiré de la canne du sucre, et le principe sucro-muqueux de quelques arbres et plantes? Celui-ci contient-il du vrai sucre, ou peut-il être converti en sucre?"

V. To remove the uncertainty which exists respecting the choice of different kinds of vinegar for different purposes, as for food, an antiseptic, various uses in manufactures, &c. and to bring the vinegar manufactory to perfection, on fixed principles, it is asked, 1. "Quelles sont les propriétés et principes différens des différentes espèces de vinaigres en usage chez nous, soit fait ici ou aporté d'ailleurs, et de quelle manière peut-on determiner facilement la force relative de différentes espèces de vinaigres, gres, sans y employer des appareils chimiques considerables ? 2. Quelles espèces de vinaigres doivent être considérées, suivant des épreuves chimiques, les plus convenables pour les differens usages, qu'on en fait ; et quelles sont les conséquences de cette théorie, qui peuvent servir au perfectionnement des trafics de vinaigre ?"

VI. "Quelle est apparemment l'origine du spermaceti, ainsi nommé ? Peut-on séparer cette substance de l'huile de baleine, ou en effectuer la production dans celle-ci ; et cette production pourra-t-elle être avantageuse ?"

VII. "Peut-on, de ci qu'on connait des principes des alimens des animaux, expliquer suffisamment l'origine des principes ou parties constituantes éloignées du corps humain, comme sont spécialement la terre calcaire, la soude, le phosphore, le fer, &c. ? Sinon, sont ils portés d'ailleurs dans le corps animal, ou y a-t-il des expériences et des observations suivant lesquelles on peut supposer qu'au moins quelques-uns de ces principes, quoiqu'on ne les puisse composer ni analyser par des moyens chimiques, sont produits par une action propre des organes vivans ?"

Should the latter opinion be adopted in the answer, it will be sufficient to prove the production of one of the aforesaid principles.

VII. "Qu'est-ce que l'experience a démontré suffisamment concernant l'accéléation de la germination des semences, que Humboldt a essayé le premier, en les arrosant de l'acide muriatique oxygéné, comme aussi concernant d'autres moyens qu'on a employés, hormis les engrais communs et la chaleur pour accélérer la vegetation des plantes en genéral, et la germination des plantes, de quelle manière ces moyens agissent ? Quel secours nous donne ce que nous en connaissons, pour des recherches ultérieures, soit des moyens déjà fait voir et confirmé, pour la culture des vegetaux utiles ?"

IX. "Jusqu'à quel point connait-on le sable mouvant (het welzand) pour autant qu'il se trouve à differens endroits de cette republique, surtout dans la proximité des côtes de la mer du Nord ? Que sait-on de son étendue et de sa profondeur ? De la nature différente, de l'epaisseur et de la variation de ses couches ? De sa mobilité ? Et de quelle maniere peut-on expliquer ce qu'on voit avoir lieu quelque fois à cet égard ? Quelles indications utiles peut-on deduire de ce que nous en savons soit en faisant des puits pour obtenir de la meilleure eau de source, soit en plaçant les fondemens pour les edifices, ecluses ou autres batimens ?"

X. The windmill being one of the most useful machines for the welfare, and even the existence of the principal part of the kingdom, the society demands, Quelle doit être la position de la toile des ailes, sur les lattes, par rapport au plan du mouvement des ailes, et à chaque distance de l'axe, afin que l'effet du moulin soit toujours le plus favorable ?"

The Society requests, 1. "Une esquisse des principales maniéres usitées chez les constructeurs des moulins selon lesquels ils mettent les lattes aux ailles. 2. Une comparaison de ces différentes manières entre elles, et surtout avec les ailles de Van Dijl, qui ont octroyées depuis quelques années. Une demonstration de la manière jugée la meilleure, fondée sur une theorie exacte, et confirmée par des épreuves."

XI. As experience has proved on the one hand, the great effect of sluices, (witwaterende sluizen) and on the other, the utility des déversoirs soirs (overlaten) for the evacuation of l'eau intérieure (binnen-water), the Society requests ; " Une théorie comparative et prouvée par des experiences, de l'action de l'un et de l'autre, comme aussi une demonstration dans quels cas on doit préférer l'un à l'autre."

XII. " Quelle est la cause que la vegetation des plantes est beaucoup mieux accélerée par la pluie que par l'arrosement avec de l'eau de pluie, de source, de rivière on de fossé ? Y-a-t-il des moyens de communiquer à ces differentes eaux cette qualité de la pluie, qui accélere la vegetation, et quels sont ces moyens ?"

XIII. " Quelles espèces de plantes graminées fournissent dans les prairies des terrains sablonneux, argilleux et marécageux, les alimens les plus nutritifs aux bêtes à cornes et aux chevaux ; et de quelle manière peut-on les cultiver et les multiplier le mieux au lieu de ces plantes, qui sont moins utiles dans ces prairies ?"

XIV. " Jusqu'à quel point peut-on juger de la fertilitè des terrains, soit cultivés ou non cultivés, par les plantes qu'on voit végéter naturellement sur ces terrains ; et quelles indications donnent-elles de ce qu'on peut ou doit faire pour l'amélioration de ces terrains ?"

XV. " Que doit on penser de la fermentation panaire ainsi dite ? Est-elle une espèce particulière de fermentation ? Quelles matieres en sont susceptibles ? Dans quelles circonstances peut-elle avoir lieu? Quels sont les phénomènes qui accompagnent cette fermentation depuis le commencement jusqu'à la fin ! Quels changemens subissent les parties constituantes les plus prochaines des corps, qui sont sujets a cette fermentation ? Et qu'apprend-on par l'un et l'autre pour perfectionner l'art de cuire le pain ?"

XVI. Que sait-on de la generation et de l'économie des poissons dans les rivières et les eaux stagnantes, surtout de ces poissons qui nous servent de nourriture ? Et que peut-on en deduire concernant ce qu'on doit éviter pour favoriser les multiplications des poissons ?"

The Society offers a prize of thirty ducats, in addition to the ordinary one, for a satisfactory answer to each of the questions, No. I, IV, V, VI, IX, X, XI, XIII, XIV, and XV.

XVII. As the chemical analysis of vegetables, notwithstanding the considerable progress which has been made in it of late years, is not yet brought to that degree of perfection that we can trust to its results in all cases, since they sometimes differ considerably after the most careful analysis ; and as the knowledge of the nature of plants, of their greater or less utility for nourishment, and of their medicinal properties, depend upon it in a great degree, the Society offers an extraordinary prize of 50 ducats, in addition to the ordinary medal, " à celui, qui par des experiences répétées ou nouvelles (qu'on aura trouvé exactes en les répetant), aura réduit l'analyse chimique des plantes au plus haut degré de perfection, et aura écrit le precis le plus parfait des procédés les plus convenables, pour faire l'analyse chimique des matières végétales en tout cas par la voie la plus simple, mais en même temps la plus certaine, de manière qu'on obtient toujours, en répétant avec soin les procédés, les mêmes resultats."

The three following questions were proposed in the preceding years, to be answered before the 1st of January, 1812.

A. Experience, especially natural history united with chemistry, having already proved in general, that in organized bodies, which differ considerably in form and external structure, a remarkable difference is observed in the constituent principles, and chemical composition; and the Society judging that botany may even acquire new light from the chemical examination of vegetables, proposes this question;

"Quel est le rapport qui existe entre la structure exterieure et la composition chimique des végétaux? Peut-on distinguer par des charactères chimiques les familles naturelles des plantes? Quels sont, dans ce cas, ces caractères? Et peuvent-ils servir à déterminer et à distinguer avec plus de certitude les familles naturelles des plantes?"

B. As the Linnean system in the classification of animals, has from time to time undergone several alterations, and as it is to be feared that the difficulties in the study of natural history will increase in proportion as the science extends, and that confusion will arise, instead of the order to which the system formerly fixed the natural history of animals, the Society proposes the following question:

"Est-ce qu'on a fait déjà assez de progrès dans cette science, pour introduire un autre système qui n'étant pas basé sur des positions gratuitement adoptées, est préférable à tous les autres, pour l'invariabilité et la simplicité des caractères, et qui mériterait pour cet effet d'être généralement adopté? Si la réponse est affirmative, quels sont les principes sur lesquels ce système est basé? Si non, quel système de ceux qui existent, est préférable pour l'état present de la science, et par quelle vois pourrait-on surmonter les difficultés susdites?"

As the consideration of this question may be extended to great length, it is necessary to observe, that short memoirs only will be admitted.

C. As it is a well established rule in agriculture, that herbs cultivated upon the same land ought to be varied, and as it is very important, both for the preservation of the fertility of such lands, and for the success of the herbs which are cultivated, that they follow in order, the Society desires: "Qu'on fasse voir par des principes physiques et chimiques, et suivant l'experience de l'agriculture, dans quel ordre où dans quelle succession les herbes qu'on cultive dans ce pays-ci sur des terrains argilleux, marécageux, sablonneux et mêlés, doivent se suivre dans le même champ, afin que leur culture se fasse avec le plus grand avantage; surtout dans quel ordre on doit cultiver les herbes pour la nourriture des bestiaux et d'autres sur des terrains sablonneux et élevés, principalement ceux qui sont nouvellement défrichés, afin de ménager autant que possible les engrais, et de prevenir l'epuissement de la fertilité des terrains?"

---

The case of the Hon. Robert Grosvenor has excited the public mind to a degree that makes the notice of it here a proper measure. In 1801 this young gentleman was vaccinated by Dr. Jenner. In the month of May last he was attacked with febrile symptoms, succeeded, on the third day, by an eruption, which had the appearance, in its early stage, of small pox. This eruption became confluent, was accompanied with fever and delirium of such violence, as to indicate great danger. On the eighth day the fever subsided, and the eruption took on appearances, unusual, if they have ever occurred, in confluent variola. At present, we we can only say, that facts have arisen in the course of this disease, which shew its progress to have been much influenced, and its character modified, by the previous vaccination. Among those who decide from evidence, opinion will certainly be suspended, until the whole of the testimony that the nature of the case admits comes fairly before the public. We know that a Report of the case is preparing, which may be expected to contain all the material facts; and upon those facts only can a rational conclusion be founded. The other children of Earl Grosvenor, who had been vaccinated, were, in consequence of this alarm, subjected to variolous inoculation, and were found to have been secured from its effects by the previous vaccination.

---

The resources with which Nature is provided for distributing the vital fluid throughout the bodies of animals, when the principal trunks of arteries are destroyed, has been remarkably exemplified in experiments lately made by Mr. Ashley Cooper. That gentleman tied the *aorta descendens* of dogs, very near to the heart, in a way to stop the current of blood passing, by that vessel, to all the lower parts of the frame. The animals seemed to sustain no great inconvenience by this; the wounds soon healed, the health was not impaired, the secretions proceeded as usual, and the creatures remained active and lively. When they were destroyed after some weeks, or months, for the purpose of ascertaining the changes that had happened, from the destruction of a part presumed to be so essential to life, the aorta was found obliterated where the ligature had been fixed, and the blood had been transmitted by anastymosing branches.

---

A marked instance of the appearance of Small-pox twice in the same person, has occurred in the case of the Rev. Mr. Rowley, son of lady Rowley. About forty years ago Mr. Rowley, then a child, was inoculated for the Small-pox, by Mr. Adair, Surgeon general; and had a considerable eruption. On the 5th of June last, he was seized with fever, and an eruption appeared on the third day: there were two hundred pustules on the face, and the distemper has proved a severe case of distinct Small-pox.

Another instance of Small-pox after inoculation has happened to Miss S. Booth, of Covent Garden Theatre. At five years of age this young lady was inoculated for Small-pox. The progress of the arm was regular, she had considerable fever, and the whole of the appearances were of a nature to afford, it was believed, a perfect security from any future attack of the disease. On the 20th of June, she was seized with febrile symptoms, which proved the precursor of Small-pox: on Sunday, the 3d day from the attack, pustules appeared on the forehead and scalp. The eruption spread to other parts of the frame, accompanied with sore throat. This eruption passed through the usual forms and stages of the disease, and constituted an undoubted case of *Variola.*

